# Medical and Surgical Report of the Presbyterian Hospital, City of New York

**Published:** 1900-10

**Authors:** 


Medical and Surgical Report of the Presbyterian Hospital, City of New York. Volume IV., January, 1900. Andrew J. McCosh, M. D., and W. Gilman Thompson, M. D., editors.

The excellent series of hospital reports of the Presbyterian Hospital of New York, instituted in 1896, is worthily continued as the volume of 1899, Volume IV., will testify. Other large hospitals are imitating the efforts of the Johns Hopkins, Massachusetts General and Presbyterian Hospitals by publishing annual reports and much valuable information regarding clinical details are finding permanent repositories.

In the volume before us are excellent articles on the surgery of the testicles, surgery of the biliary passages, cases of subphrenic abscess, scleroderma, empyemia, suppurative hepatitis, heart murmurs and heart lesions, statistics of 100 cases of cancer of the breast, pneumothorax, penetrating bullet wound of the chest and a paper on recording and classifying surgical histories.

These papers are all well written and some beautifully illustrated, especially the article on Surgery of the Testicle, by Dr. Eliot, Jr.

